# Protective Role of Enalapril in Anthracycline-Induced Cardiotoxicity: A Systematic Review

**DOI:** 10.3389/fphar.2020.00788

**Published:** 2020-05-27

**Authors:** Yili Zhang, Junjie Liu, Yuan Li, Nannan Tan, Kangjia Du, Huihui Zhao, Juan Wang, Jian Zhang, Wei Wang, Yong Wang

**Affiliations:** ^1^School of Traditional Chinese Medicine, Beijing University of Chinese Medicine, Beijing, China; ^2^Dongzhimen Hospital, Beijing University of Chinese Medicine, Beijing, China; ^3^Ministry of Education Key Laboratory of TCM Syndrome and Formula & Beijing Key Laboratory of TCM Syndrome and Formula, Beijing, China; ^4^School of Life Science, Beijing University of Chinese Medicine, Beijing, China

**Keywords:** cardioprotection, onco-cardiology, chemotherapy, evidence-based medicine, anthracycline

## Abstract

**Background:**

Evidence of the preventive and therapeutic effects of enalapril on cardiotoxicity caused by chemotherapy needs to be further confirmed and updated.

**Methods:**

We performed a systematic review of studies from electronic databases that were searched from inception to January 29, 2019, and included relevant studies analyzing enalapril as a cardioprotective agent before or during the use of anthracyclines by oncology patients. Homogeneous results from different studies were pooled using RevMan 5.3 software. The Cochrane risk-of-bias tool was used to determine the quality of the studies.

**Results:**

We examined and screened 626 studies according to specific criteria and ultimately included seven studies that were relevant to the indicated topic. Among them, three studies reported the incidence of death during 6- and 12-month follow-up periods. Six of the seven included studies showed possible positive results, suggesting that enalapril plays a cardioprotective role, while five of these studies showed that there was a significant difference in the left ventricular ejection fraction (LVEF) between an enalapril group and a control group (weighted mean difference (WMD) = 7.18, 95% CI: 2.49–11.87, I^2^ = 96%, P < .001). Moreover, enalapril was beneficial in reducing troponin I (TnI), creatine kinase myocardial band (CK-MB) and N-terminal pro-b-type natriuretic peptide (NT-proBNP) levels in cancer patients treated with anthracycline.

**Conclusions:**

Although a protective effect of enalapril on myocardial toxicity was observed in terms of the LVEF values and TnI, CK-MB and NT-proBNP levels, its use in the prevention and treatment of cardiotoxicity caused by anthracycline needs to be investigated by more scientific research.

## Introduction

With the rapid increase in the global population and the development of an aging society, cancer is becoming increasingly prominent as a leading cause of death (GBD 2016 Causes of Death Collaborators, 2017). According to statistics from the International Agency for Research on Cancer, there were an estimated 18.1 million new cancer cases and 9.6 million cancer-related deaths in 2018 (Rebecca et al., 2018). Nevertheless, the great progress in therapeutic strategies for various tumors has led to a longer life and a higher quality of life (Rohit and Yeh, 2016), which has enabled observations of the side effects of anticancer therapy and increased morbidity and mortality from other causes.

The antibiotic anthracycline (represented by doxorubicin (DOX)) is highly effective and is currently the most commonly used chemotherapeutic drug for various cancers, including leukemia, solid tumors, soft tissue sarcomas and breast cancer (Damiani et al., 2016; Songbo et al., 2019). However, anthracycline-related cardiac toxicity is reportedly as high as 57%, and the mortality rate from these heart diseases is reportedly 8.2 times higher than that in normal persons (Brewster et al., 2014), substantially limiting its clinical application. Only one drug, dexrazoxane, is approved by the US Food and Drug Administration (FDA) to be indicated for contributing a certain protective effect in patients with cardiotoxicity; however, its use is limited to patients receiving a high cumulative dose of anthracyclines (Tomlinson et al., 2019). In July 2011, the FDA released a declaration restricting the use of dexrazoxane to adult patients with cancer who receive >300 mg/m^2^ doxorubicin (an anthracycline) or >540 mg/m^2^ epirubicin (another chemotherapeutic agent) and have general approval for the use of dexrazoxane for cardioprotection (Tebbi et al., 2007; Salzer et al., 2010). Furthermore, several previous studies have shown that dexrazoxane may increase the incidence of myelodysplastic syndrome and secondary cancers (Tebbi et al., 2007). The Committee for Medicinal Products for Human Use (CHMP) in the UK even recommended a few restrictions on the use of dexrazoxane in both children and adults with cancer (EMA, 2019). Therefore, there is an urgent need to identify the underlying mechanism and novel therapeutic agents that can prevent and/or reverse cancer treatment-induced cardiovascular adverse effects.

Angiotensin-converting enzyme inhibitors (ACEIs) (Cardinale et al., 2006; Zamani et al., 2018) are considered promising cardioprotective agents that can be used for cardiac protection during chemotherapy. The mechanism of cardiac protection is mainly related to the SDF-1a/CXCR4 axis (Wen et al., 2012), hypoxia-inducible factor-1a (HIF-1a) upregulation (Luft, 2017; Zhang and Lin, 2010), nuclear factor kappa-light-chain-enhancer of activated B cells axis (Puddighinu et al., 2018) and a decrease in ROS (Heusch, 2012).

At present, some new clinical trials and meta-analyses (Conway et al., 2015; Gupta et al., 2018) assessing the use of ACEIs for anthracycline-induced cardiotoxicity have been published and may provide higher quality evidence suggesting that ACEIs are effective as cardioprotective agents. Given that existing evidence, therefore, we have the opportunity to perform this systematic review of randomized controlled trials (RCTs) to expand and update knowledge of cardioprotective role of ACEIs on anthracycline-induced cardiotoxicity. We hope that the findings of this study strengthen the evidence of the effectiveness of enalapril with regard to the prevention and treatment of anthracycline-induced cardiotoxicity.

## Materials and Methods

This study was registered at PROSPERO (registration number: CRD42019124671; http://www.crd.york.ac.uk/PROSPERO). This meta-analysis was conducted based on the Preferred Reporting Items for Systematic Reviews and Meta-analysis (PRISMA) criteria.

### Types of Studies

We included all prospective RCTs focusing on enalapril as a strategy for the treatment of cardiotoxicity caused by anthracycline. Crossover trials, quasi-RCTs, animal experiments and other studies published repeatedly or without access to complete data were excluded.

### Types of Participants

Participants who accepted conventional chemotherapy were eligible. All participants were included in this review regardless of age, race, sex and cancer type.

### Types of Interventions

We only included studies in which interventions, including enalapril alone or combined with other agents, were used to prevent the toxic effects of anthracycline on the heart regardless of duration or dosage.

### Types of Comparisons

Control groups that could be used to show the cardioprotective role of enalapril were considered.

### Types of Outcomes

The primary outcomes were death from any cause and changes in the left ventricular ejection fraction (LVEF) measured by conventional echocardiographic parameters. The secondary outcomes mainly focused on conventional echocardiographic parameters (except for LVEF), cardiac biomarkers (plasma brain natriuretic peptide, plasma myocardial enzyme, and troponin I (TnI) levels) and adverse events.

### Information Sources and Search Strategy

A comprehensive search strategy was carried out that included searches of PubMed/Medline (from inception to January 2019), EMBASE (from inception to January 2019), the Cochrane Library (from inception to January 2019) and ClinicalTrials.gov (from inception to January 2019). The studies that met the inclusion criteria were searched. The following search terms were searched individually or jointly at the time of retrieval: ‘enalapril’, ‘ace-inhibitor’, ‘angiotensin-converting enzyme inhibitors’, ‘ace inhibitor’, ‘angiotensin converting enzyme inhibitors’, ‘angiotensin-converting enzyme antagonists’, ‘angiotensin converting enzyme antagonists’, ‘ACE inhibitors’, ‘cancer’, ‘tumor’, ‘malignant’, and ‘solid tumor’. Only studies published in English were considered. NoteExpress 3.0 Software was used to manage the literature.

### Study Selection and Study Quality Assessment

Two reviewers independently screened the literature and recorded the reasons for exclusion. At the time of data extraction, a “table of characteristics” was generated to extract information regarding the included trials, including the author, age of the participants, diagnostic criteria, sample size of the experimental group and control group, intervention applied to the two groups, outcomes and adverse events. The methodological quality of the RCTs was assessed independently per the Cochrane Handbook for Systematic Review of Interventions, Version 5.1.0., including randomness, blindness, outcome reporting and other bias. The evaluation degree of each item was divided into the following three grades: low bias risk, high bias risk and unclear bias risk. The evaluation of the methodological quality was performed independently by two reviewers, and discrepancies were solved through mutual consensus.

### Data Analysis

RevMan 5.3 software was used for the meta-analytic calculations. The Q test was conducted to estimate the total percentage of variation in each study derived from heterogeneity rather than chance, and the I^2^ statistic was used to quantify the heterogeneity. The model used to synthesize the data needed to consider the existence and degree of heterogeneity. For instance, if the I^2^ statistic was less than 50% and the P-value was more than 0.1, the fixed-effects model was chosen. If the P-value was less than 0.1, the treatment effects were calculated with a random-effects model. Random effect models were used for the subgroup analysis and when significant heterogeneity existed among the studies. According to the Cochrane Handbook version 5.1.0, a random-effects meta-analysis model involves an assumption that the effects being estimated in the different studies are not identical but follow some distribution. The model represents our lack of knowledge about why real, or apparent, intervention effects differ by considering the differences as if they were random. The center of this distribution describes the average of the effects, while its width describes the degree of heterogeneity.

For the dichotomous variables, the pooled relative risk (RR) and 95% CI were used as the effect measures. For the continuous outcomes, the weighted mean difference (WMD) was used when the units of the outcomes were the same, while the standardized mean difference (SMD) was used when the units and/or measurement methods of the outcomes were inconsistent. If fewer than two studies reported the same results or the heterogeneity among the studies was obvious, the results of our systematic review are narratively reported.

### Subgroup Analysis and Sensitivity Analysis

To solve the problems of heterogeneity and secondary analysis, the subgroup analysis was very important. A sensitivity analysis was also implemented from the methodological, statistical and clinical aspects to explore potential sources of heterogeneity. When the results of different experiments greatly varied and the heterogeneity test showed significant differences, we removed one trial that significantly differed from the other trials (due to clinical, methodological, or other factors) and then combined the remaining studies to compare the before and after results. For any meta-analysis involving 10 or more studies, we used funnel diagrams to assess the possibility of publication bias (Kakia et al., 2016).

## Results

### Search Results and Study Characteristics

**Figure 1:**
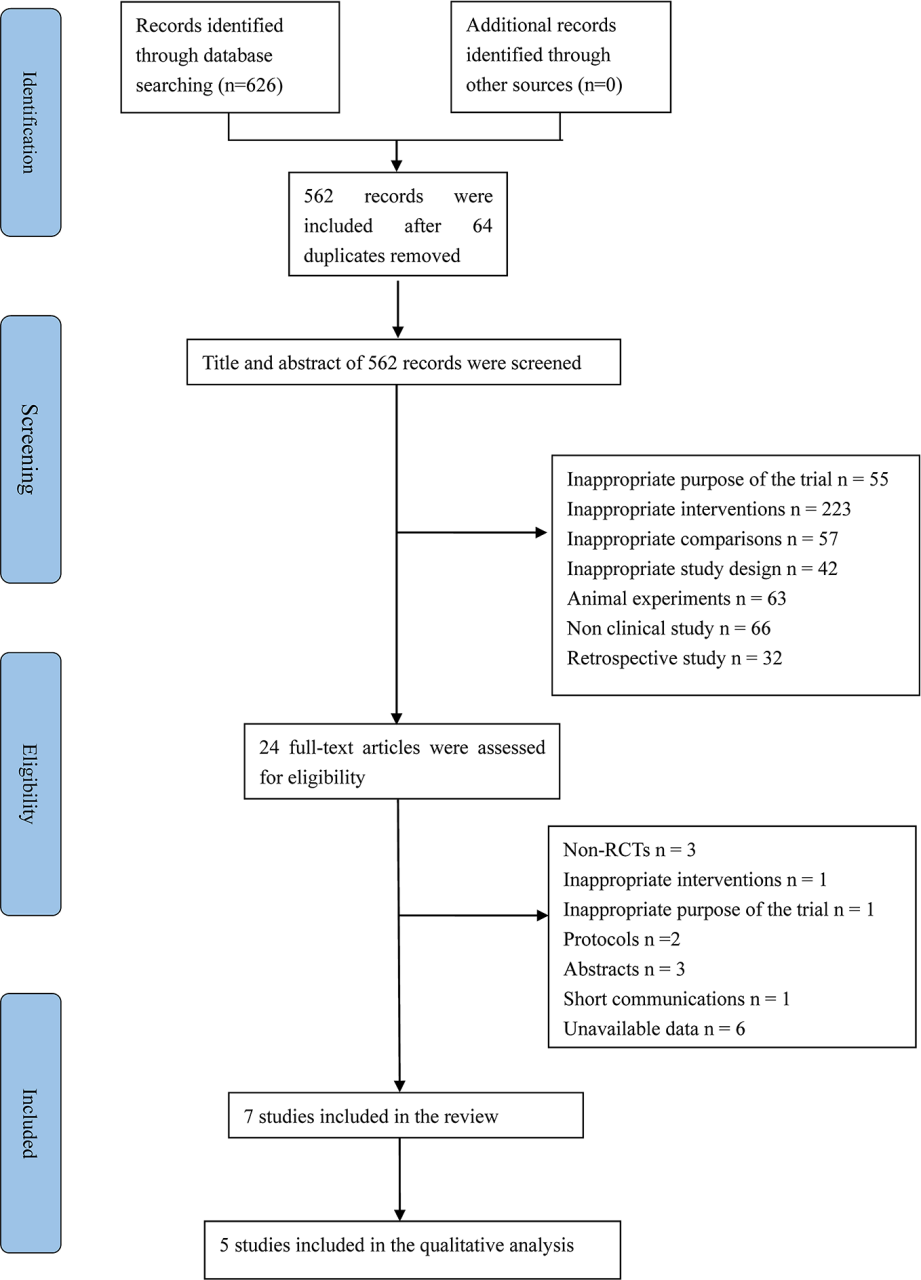
PRISMA flow diagram. RCTs, randomized controlled trials; PRISMA, Preffered Reporting Items for Systematic review and Meta-Analysis.

Initially, the literature search yielded 626 citations concerning enalapril for the treatment or prevention of cardiotoxicity from an electronic database (**Figure 1**). After the two reviewers screened the title, abstract and full text of each citation according to the inclusion criteria, 619 articles were excluded as duplicates, non-RCTs, reviews, retrospective studies or studies with objectives that differed from the aim of this review. Ultimately, seven RCTs were included, and we analyzed the quantitative data reported in five studies.

In total, 848 participants were included in this systematic review. Of these participants, 382 were treated with enalapril. In addition, 137 patients in Cardinale’s study (Cardinale et al., 2018) started taking enalapril only during or after an increase in troponin was evident during chemotherapy. Forty-five patients in one trial (Bosch et al., 2013) received a combination treatment of enalapril and carvedilol. The trials included patients with pediatric cancer, breast cancer, lymphoma, multiple myeloma, lung cancer and other malignancies. The baseline LVEF was comparable between the experimental and control groups in all studies. The duration of follow-up in the selected studies ranged from 6 to 36 months. Seven trials were conducted in different countries, including the US, Italy, Greece, Spain, Iran, and India. The details of the characteristics of the seven included trials are provided in **Table 1**.

**Table 1:** Characteristics of the included trials.

Study ID	Sample size (EG/CG)	Median age	Types of cancer	Patients and detailed chemotherapy or radiotherapy	Intervention		Baseline LVEF		Follow-up duration	Outcomes
					EG	CG	EG	CG		
Silber et al., 2004	69/66	EG: 17.8 ± 5.60 CG: 18.9 ± 6.17	Long-term survivors of pediatric cancers	The target population consisted of patients aged 8 years and older who developed cancer before the age of 20 years and had been treated with anthracyclines.	Enalapril	Placebo	NR	NR	Mean follow-up duration of 34.6 months	The rate of change in the MCI and LVESWS, stress-velocity index, left ventricular shortening fraction, adverse events, functional status, and quality of life
Cardinale et al., 2006	56/58	45 ± 12	Breast cancer, acute myeloid leukemia, etc.	High-dose chemotherapy including carmustine, etoposide, cytarabine, melphalan, daunorubicin, carboplatin, idarubicin, mitoxantrone, epirubicin, etc.	Enalapril	None	NR	NR	12 months	The occurrence of cardiotoxicity, efficacy of enalapril on LVEF, and adverse cardiac events
Georgakopoulos et al., 2010	43/40	EG: 47.4 ± 16.2 CG: 49.1 ± 19.4	Lymphoma	The CT regimen consisted of 6–8 cycles of the “ABVD schema” for HL as follows: doxorubicin (25 mg/m^2^), bleomycin (10 mg/m^2^), vinblastine (6 mg/m^2^), and dacarbazine (375 mg/m^2^) intravenously on day 1 and day 15 every 4 weeks. The NHL patients received the “R-CHOP schema” as follows: rituximab (375 mg/m^2^), cyclophosphamide (750 mg/m^2^), doxorubicin (50 mg/m^2^), and vincristine (1.4 mg/m^2^) intravenously on day 1 and prednisolone (100 mg/m^2^) orally on days 1–5 every 3 weeks.	Enalapril	None	65.2 ± 7.1	67.6 ± 7.1	36 months	Echocardiographic evaluations
Bosch et al., 2013	45/45	50 ± 13	Acute leukemia, relapsed or refractory; Hodgkin’s and non-Hodgkin’s lymphoma; and multiple myeloma	NR	Enalapril and carvedilol	None	NR	NR	6 months	Global LVEF, TnI and BNP levels, incidence of death, heart failure or significant LVSD, diastolic function, and incidence of severe life-threatening adverse events
Janbabai et al., 2017	34/35	EG: 47.76 ± 11.81 CG: 47.06 ± 12.39	Breast cancer (60 patients), Hodgkin’s lymphoma (six patients), Wilms tumor (one patient), lung cancer (one patient) and bone sarcoma (one patient)	Sixty patients had breast cancer and received doxorubicin and cyclophosphamide; six patients had Hodgkin’s lymphoma and underwent R-CHOP chemotherapy, which included rituximab, cyclophosphamide, doxorubicin, vincristine, and prednisolone; one patient had a Wilms tumor and received vincristine, dactinomycin, doxorubicin, cyclophosphamide, and etoposide; one patient had lung cancer and received vincristine, doxorubicin, and cyclophosphamide; and one patient had bone sarcoma and received cisplatin and doxorubicin. All patients received doxorubicin, and most patients received cyclophosphamide. None of the patients received trastuzumab or radiotherapy during the 6-month follow-up period.	Enalapril	Placebo	59.39 ± 6.95	59.61 ± 5.70	6 months	Changes from baseline in LVEF, troponin I and CK-MB levels and the incidences of death, HF, significant LV systolic dysfunction, diastolic dysfunction, and severe life-threatening adverse events
Cardinale et al., 2018	136/137	51 ± 12	Breast cancer; acute leukemia, non-Hodgkin’s lymphoma; Hodgkin’s lymphoma; lymphoma, unspecified type; sarcoma; etc.	The median number of cycles of anthracyclines was four cycles delivered over 65 days. Epirubicin and doxorubicin were the most commonly prescribed anthracyclines with a median cumulative dose of 360 and 240 mg/m^2^, respectively. In total, 63% of the patients with breast cancer were treated with taxanes, and 22.5% of the patients were treated with trastuzumab. In total, two patients were treated with a tyrosine-kinase inhibitor, imatinib.	Enalapril in all patients was started before chemotherapy	Enalapril started only in patients with an increase in troponin during or after chemotherapy	63.5 ± 5.9	63.9 ± 6.1	12 months	An incidence of troponin elevation above the threshold, LVEF <50% and >10% LVEF reduction, death from cardiovascular causes, death from any cause, hospitalization for cardiovascular causes, and major adverse cardiovascular events
Gupta et al., 2018	44/40	EG: 8.85 ± 3.15 CG: 8.77 ± 2.86	Acute lymphoblastic leukemia/lymphoma	Projected cumulative anthracycline dose was ≥200 mg/m^2^.	Enalapril	Placebo	65.73 ± 5.41	64.85 ± 4.94	6 months	LVEF, changes in cardiac biomarkers, and the development of heart failure or arrhythmias

CG, control group; CK-MB, creatinine kinase-MB; EG, experimental group; NR, not reported; FS, fractional shortening; LVESWS, left ventricular end-systolic wall stress; MCI, maximum cardiac index; HL, Hodgkin lymphoma; NHL, non-Hodgkin lymphoma; HF, heart failure; LA, left atrium; LVEDD, left ventricular end-diastolic dimension; LVEDS, left ventricular end-systolic dimension; LVEDV, left ventricular end-diastolic volume; LVEF, left ventricular ejection fraction; LVESV, left ventricular end-systolic volume; LVSD, left ventricular systolic dysfunction.

### Methodological Quality

**Figure 2:**
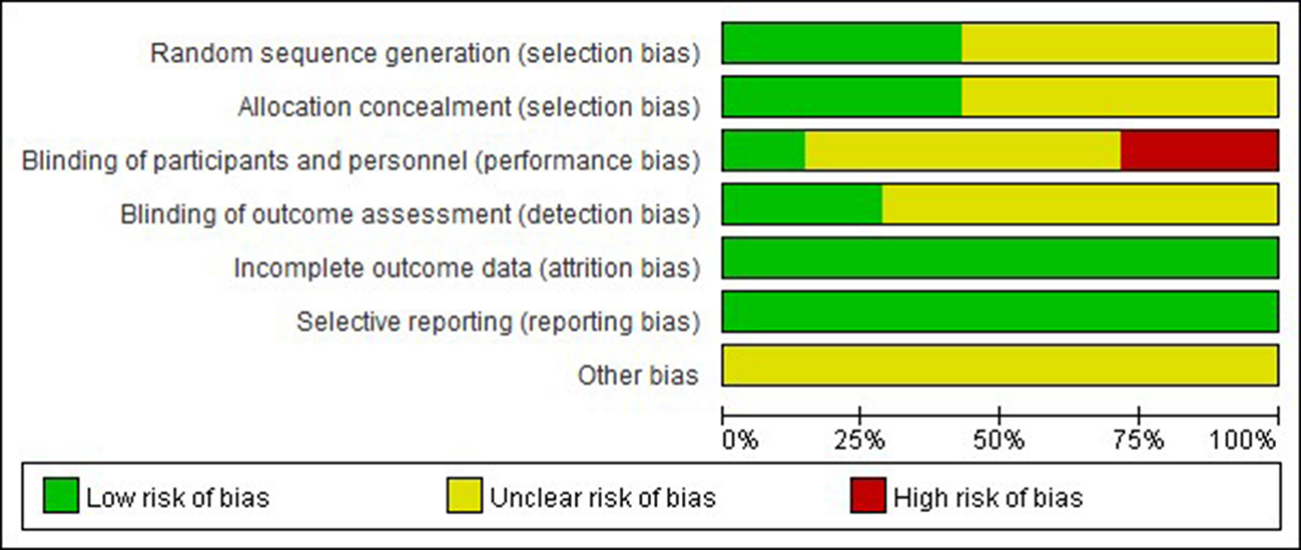
Risk of bias graph.

Regarding random sequence generation, three studies (Cardinale et al., 2006; Bosch et al., 2013; Cardinale et al., 2018) were conducted with appropriate randomization based on numbers generated with an electronic computer. The other trials only briefly mentioned ‘random’ without providing a detailed description of the specific method. Three studies (Cardinale et al., 2006; Bosch et al., 2013; Cardinale et al., 2018) described the details of the allocation concealment (using central dispensation or numbered envelopes). Two open-labeled trials (Cardinale et al., 2006; Cardinale et al., 2018) were classified as ‘high risk’ in terms of blinding. Except for the studies by Silber et al. (2004), Janbabai et al. (2017) and Gupta et al. (2018), the other studies did not report the methods used to blind the participants, researchers or outcome assessments. All studies claimed to have good baseline consistency with a trial registration number, and the attrition in both groups seemed balanced such that incomplete outcome data and selective reporting were deemed to be at a low risk. Additionally, none of the studies mentioned the other bias items. A risk-of-bias graph is shown in **Figure 2**.

### Effects of Therapy

#### Death From **A**ny Cause

Three studies (Bosch et al., 2013; Janbabai et al., 2017; Cardinale et al., 2018) reported the incidence of death during 6- and 12-month follow-up periods. Considering the potential clinical heterogeneity of the interventions (enalapril plus carvedilol vs. no treatment; enalapril vs. placebo; and enalapril in all patients started before chemotherapy vs. enalapril started only in patients with an increase in troponin during or after chemotherapy) and the different characteristics of the participants, these studies were examined as separate individual studies in the assessment. During the study implementation (Bosch et al., 2013), 11 patients were excluded from the study due to their deaths (four cancer-related deaths and seven infection-related deaths). In the ICOS-ONE trial (Cardinale et al., 2018), 10 patients died (3.7%) during the 1-year follow-up period, including eight patients in the experimental group and two patients in the control group. These deaths were all due to non-cardiovascular causes and were related to cancer progression (70%) or infection (30%). However, none of the patients died during the follow-up period in Janbabai’s study (Janbabai et al., 2017) in which the patients seemed to have a better risk control state. In addition, this finding may be related to the regimen and duration of chemotherapy.

Therefore, based on the studies examined, no conclusion can be drawn regarding the influence on cardiac-related mortality.

#### Changes in Cardiac Function: LVEF Value

**Figure 3:**
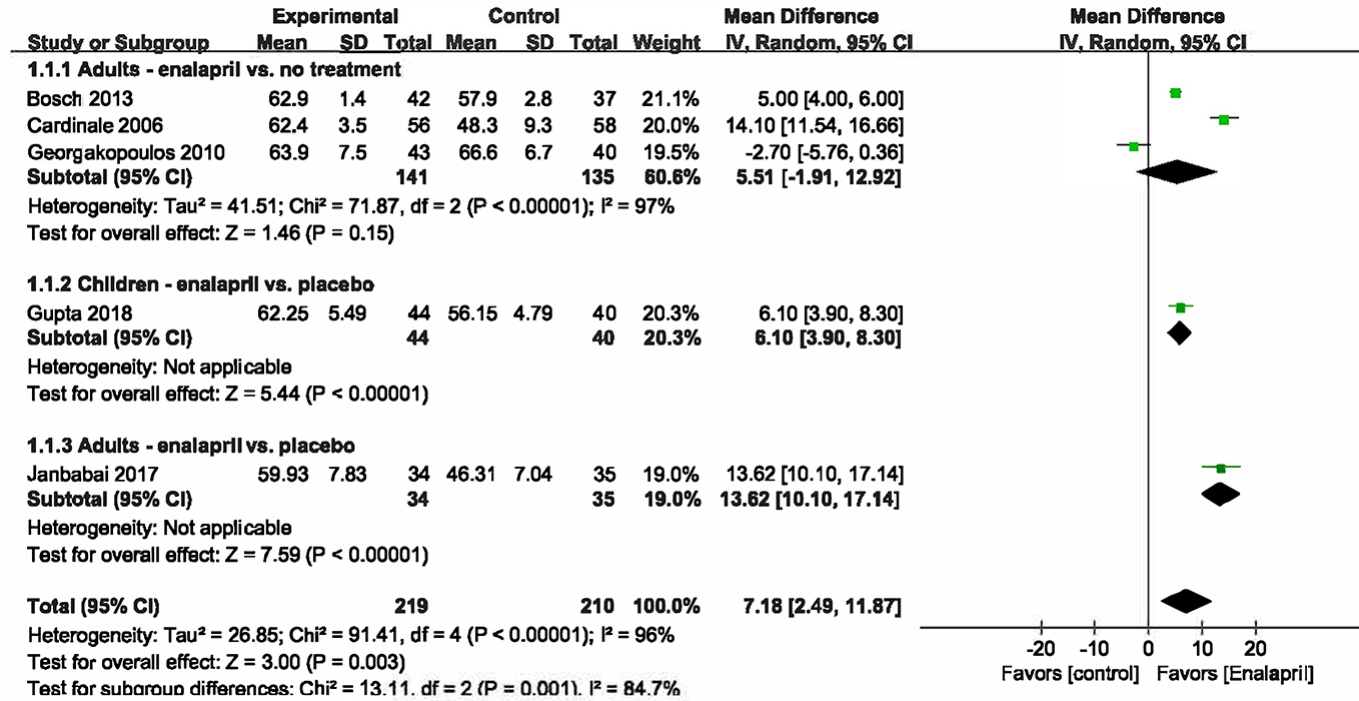
Meta analysis for LVEF value.

Five studies (Cardinale et al., 2006; Georgakopoulos et al., 2010; Bosch et al., 2013; Janbabai et al., 2017; Gupta et al., 2018) reported changes in the LVEF values *via* different control measurements. Of these studies, three studies (Cardinale et al., 2006; Georgakopoulos et al., 2010; Bosch et al., 2013) combined enalapril with no treatment, and each of the other two studies had different participants. Therefore, we combined the three studies into a subgroup while separately calculating the effects of the other two studies to generate an overall meta-analysis. The data of the five combined studies showed that the LVEF value in the intervention group after chemotherapy was significantly higher than that in the control group (WMD = 7.18, 95% CI: 2.49–11.87, P < .001) (**Figure 3**). However, substantial heterogeneity still existed among the studies after the subgroup analysis. The sensitivity analysis found that the results of one study (Georgakopoulos et al., 2010) contradicted those of the other studies, which affected the robustness of the pooling effect. After excluding this study, there was no significant change in the LVEF value compared to the original result. Furthermore, a tendency toward the opposite result did not occur when any of the studies were excluded, indicating that the stability of the current results is trustworthy.

#### Conventional Echocardiographic Parameters (Other Than LVEF)

Three RCTs (Cardinale et al., 2006; Georgakopoulos et al., 2010; Janbabai et al., 2017) evaluated the morphology and function of the heart by conventional echocardiography, but the selection of evaluation indexes was inconsistent. A meta-analysis could only be performed on the E/A index but showed no statistically significant differences between the two groups. A summary of the conventional echocardiographic parameters is provided in **Table 2**.

**Table 2:** Summary of the conventional echocardiographic parameters reported (other than the LVEF).

Parameter	Studies	WMD (95% CI)	*P-*value	P of heterogeneity	I^2^
EDV	Cardinale et al., 2006	−3.10 [−12.65, 6.45]	0.52	–	–
ESV	Cardinale et al., 2006	15.90 [9.90, 21.90]	P < 0.00001	–	–
LVEDD	Georgakopoulos et al., 2010	0.20 [−0.02, 0.42]	0.07	–	–
LVESD	Georgakopoulos et al., 2010	0.20 [0.01, 0.39]	0.04	–	–
FS %	Georgakopoulos et al., 2010	−1.60 [−3.82, 0.62]	0.16	–	–
E/A, ratio	Georgakopoulos et al., 2010; Janbabai et al., 2017	0.00 [−0.11, 0.11]	1.00	1.00	0%
E/E a	Georgakopoulos et al., 2010	−0.30 [−0.91, 0.31]	0.33	–	–
LVEDV (cm^3^)	Janbabai et al., 2017	−10.65 [−19.57, −1.73]	0.02	–	–
LVESV (cm^3^)	Janbabai et al., 2017	−19.39 [−25.56, −13.22]	P < 0.00001	–	–
LA	Janbabai et al., 2017	−0.07 [−0.25, 0.11]	0.45	–	–
AR (m/s)	Janbabai et al., 2017	−0.02 [−0.06, 0.02]	0.33	–	–

AR, aortic regurgitation; EDV, end-diastolic volume; ESV, end-systolic volume; FS, fractional shortening; LA, left atrium; LVEDD, left ventricular end-diastolic dimension; LVEDS, left ventricular end-systolic dimension; LVEDV, left ventricular end-diastolic volume; LVESV, left ventricular end-systolic volume.

#### Cardiac Biomarkers: Troponin I

An Italian trial (Cardinale et al., 2006) reported that compared with the ACEI group, a percentage of patients in the control group showed an increased TnI value during follow-up, and the mean TnI value was higher in the control group (WMD = −0.02, 95% CI: −0.04 – −0.00, P =.01). Bosch’s trial (Bosch et al., 2013) demonstrated no statistically significant differences between the two groups in the incidence of troponin I elevation at the end of or soon after a cycle of chemotherapy. One pediatric study (Gupta et al., 2018) showed elevated cTnI levels at 6 months in both groups, whereas the cTnI levels in the placebo group were significantly higher than those in the enalapril group.

#### Other Biomarkers

One study (Bosch et al., 2013) reported the b-type natriuretic peptide (BNP) levels after 6 months of follow-up. However, the results suggested that there were no significant differences between the two groups when the BNP levels were >80 ng/l or >200 ng/l. Another study (Janbabai et al., 2017) showed that enalapril reduced the creatine kinase myocardial band (CK-MB) levels more favorably using a Mann–Whitney U-test (control group median = 20.27 ng/ml, 95% CI: 18.75–21.25; enalapril group median = 16.44 ng/ml, 95% CI: 15.46–18.75, P =.006). The only pediatric study (Gupta et al., 2018) in this analysis reported proBNP and CK-MB levels. There was a significant difference in the proBNP level at 6 months (49.60 ± 35.97 vs. 98.60 ± 54.24, P < .001) between the two groups, but no difference was found in the levels of CK-MB (P =.08).

### Adverse Events

Five included studies (Silber et al., 2004; Cardinale et al., 2006; Bosch et al., 2013; Janbabai et al., 2017; Cardinale et al., 2018) described adverse events during the trial. Silber’s study reported that the side effects of enalapril include dizziness or low blood pressure (22% vs. 3% in the placebo group; P < .001) and fatigue (10% vs. 0%; P =.01). In another study, in total, 31 cardiac adverse events occurred during follow-up. Overall, the number of events, including sudden death, cardiac death, and acute pulmonary edema, in the control group was higher than that in the enalapril group. Bosch’s trial showed that nine patients and 15 patients in the intervention group and control group, respectively, had life-threatening adverse events due to sepsis. The results of the other two studies showed that safety was relatively good. One study did not find any adverse events possibly because the participants had a more favorable risk profile. Another study found that only 15% of the entire population stopped treatment with the drug, and no serious adverse drug reactions (ADRs) were reported.

## Discussion

Based on the results of different individual original studies, regarding the LVESV value, the rate of change in the left ventricular end-systolic wall stress (LVESWS), and troponin I, proBNP and CK-MB levels, enalapril still has a protective effect on the chemotherapy cycles of cancer patients. However, a conclusion regarding whether angiotensin antagonist-based prevention translates into a reduction in adverse events cannot be clearly drawn from our study, although the incidence of cardiac events in the general analysis was nominally better in the prevention group.

The current work is a comprehensive systematic review focusing on the use of enalapril in the treatment or prevention of cardiotoxicity. Prior to this, some systematic reviews have described the role of ACEIs as preventive agents for health problems, such as heart failure (Turgeon et al., 2019) and hypertension (Dimou et al., 2018). Over the past decade, cardiologists have carried out multiple small clinical trials with drugs typically used for heart failure therapy, such as ACEIs, to provide either primary or secondary prevention for anthracycline-induced cardiotoxic effects (Kaya et al., 2013). These studies have shown benefits; however, the short-term benefits may be due to hemodynamic changes rather than real heart protection. Therefore, larger and longer-term clinical studies are needed to demonstrate the true efficacy of these drugs. In addition, to meet the needs of an increasing number of cancer survivors, new insight based on mechanistic research or genetic discovery is needed to pave the way for better prevention, diagnosis and treatment of cardiovascular complications caused by cancer treatment.

In recent years, strain imaging based on echocardiography has been used identify early subclinical changes in left ventricular systolic function during cancer treatment. A number of studies have shown that global longitudinal strain (GLS) can be used as a predictor of cardiotoxicity and can detect early declines in ventricular mechanics prior to an overt reduction in LVEF (El-Sherbeny et al., 2019; Arciniegas Calle et al., 2018; Potter and Marwick, 2018). In clinical practice, GLS can also help reconcile the significance of asymptomatic fluctuations in LVEF, which occur during serial imaging (Robert et al., 2019). However, it is regrettable that there is still a lack of studies that have evaluated the effect of enalapril on GLS. Further investigation is needed to determine whether the inclusion of GLS measurements in current clinical practice will improve cardiac outcomes among patients receiving cardiotoxic cancer therapy.

Although the association between cardiotoxicity and the use of anthracyclines has been known for many decades, five of the seven studies included in this review were published in the last decade, demonstrating the increased interest in this topic in recent years. However, since all studies were single-center studies and the sample sizes were small, the generalizability of these studies is limited despite the similar results obtained by most groups. In addition, although more than two major databases were searched to identify published studies, there was no guarantee that all studies that meet the inclusion criteria were retrieved for this systematic review. Additionally, Bosch’s study used carvedilol in combination with enalapril as an intervention; thus, the efficacy of enalapril could not be analyzed separately. However, given the scientific value and methodological robustness of the study, we included this study in the evaluation.

ACEIs are widely used in the clinic because of their class effect. The reason we focused on enalapril in this study was that different ACEIs may involve combinations of drugs, indications and applicable objects in clinical practice. The ultimate purpose of this study was to provide high-quality evidence to inform clinical decision making regarding specific drugs. If all the specific drugs had been included in the analysis, the extrapolation of the study results may not have been accurate. However, if any new study is carried out in the future, it will be meaningful to conduct a comprehensive analysis of the class effect. Furthermore, the duration of treatment in this work may limit the generalizability of the results. Most studies did not analyze the persistence of left ventricular dysfunction, and the patients were followed up for only 6 months. Long-term results may enhance scientific consistency in the use of ACEIs in this setting and may have the potential to demonstrate sustained and lasting benefits. Although the research carried out to date has been excellent and the preliminary results are considered satisfactory, normative research with sufficient robustness to indicate the routine use of ACEIs to prevent cardiotoxicity induced by anthracycline drugs is still lacking.

## Author Contributions

YW and WW conceived the idea for this study. YZ and YL conducted the search. JL, NT, and KD interpreted the data and drafted the figures. YZ, YL, and HZ drafted the manuscript. JW and JZ conducted the quality check on inclusion criteria. YZ and NT performed the statistical tests. All authors read and approved the final manuscript.

## Funding

This study was funded by the National Natural Science Foundation of China (81822049, 81673712, 81673802), Beijing Nova program (Z171100001117028), Fok Ying Tung Education Foundation (151044), and the National Key R&D Program of China (2017YFC1700100, 2017YFC1700102), The funders had no role in the study design, data collection and analysis, the decision to publish, or the preparation of the manuscript.

## Conflict of Interest

The authors declare that the research was conducted in the absence of any commercial or financial relationships that could be construed as a potential conflict of interest.

## References

[B1] Arciniegas Calle M. C. Sandhu N. P. Xia H. Cha S. S. Pellikka P. A. Ye Z. (2018). Two-dimensional speckle tracking echocardiography predicts early subclinical cardiotoxicity associated with anthracycline-trastuzumab chemotherapy in patients with breast cancer. BMC Cancer 18, 1037. 30359235 10.1186/s12885-018-4935-z PMC6203211

[B2] Barbosa R. R. Bourguignon T. B. Torres L. D. Arruda L. S. Jacques T. M. Serpa R. G. (2018). Anthracycline-associated cardiotoxicity in adults: systematic review on the cardioprotective role of beta-blockers. Rev. Assoc. Med. Bras. 64, 745–754. 10.1590/1806-9282.64.08.745 30673046

[B3] Bosch X. Rovira M. Sitges M. Domènech A. Ortiz-Pérez J. T. de Caralt T. M. (2013). Enalapril and carvedilol for preventing chemotherapy-induced left ventricular systolic dysfunction in patients with malignant hemopathies: the OVERCOME trial. J. Am. Coll. Cardiol. 61, 2355–2362. 10.1016/j.jacc.2013.02.072 23583763

[B4] Brewster D. H. Clark D. Hopkins L. Bauer J. Wild S. H. Edgar A. B. (2014). Subsequent hospitalisation experience of 5-year survivors of childhood, adolescent, and young adult cancer in Scotland: a population based, retrospective cohort study. Br. J. Cancer 110, 1342–1350. 10.1038/bjc.2013.788 24366296 PMC3950849

[B5] Cardinale D. Colombo A. Sandri M. T. Lamantia G. Colombo N. Civelli M. (2006). Prevention of high-dose chemotherapy–induced cardiotoxicity in high-risk patients by angiotensin-converting enzyme inhibition. Circulation. 114, 2474–2481. 10.1161/CIRCULATIONAHA.106.635144 17101852

[B6] Cardinale D. Ciceri F. Latini R. Franzosi M. G. Sandri M. T. Civelli M. (2018). Anthracycline-induced cardiotoxicity: A multicenter randomised trial comparing two strategies for guiding prevention with enalapril: The International CardioOncology Society-one trial. Eur. J. Cancer 94, 126–137. 10.1016/j.ejca.2018.02.005 29567630

[B7] Conway A. McCarthy A. L. Lawrence P. Clark R. A. (2015). The prevention, detection and management of cancer treatment-induced cardiotoxicity: a meta-review. BMC Cancer 15, 366. 10.1186/s12885-015-1407-6 25948399 PMC4427936

[B8] Damiani R. M. Moura D. J. Viau C. M. Caceres R. A. Henriques J. A. P. Saffi J. (2016). Pathways of cardiactoxicity: comparison between chemotherapeutic drugs doxorubicin and mitoxantrone. Arch. Toxicol. 90, 2063–2076. 10.1007/s00204-016-1759-y 27342245

[B9] Dimou C. Antza C. Akrivos E. Doundoulakis I. Stabouli S. Haidich A. B. (2018). A systematic review and network meta-analysis of the comparative efficacy of angiotensin-converting enzyme inhibitors and angiotensin receptor blockers in hypertension. J. Hum. Hypertens 33, 188–201. 10.1038/s41371-018-0138-y 30518809

[B10] El-Sherbeny W. S. Sabry N. M. Sharbay R. M. (2019). Prediction of trastuzumab-induced cardiotoxicity in breast cancer patients receiving anthracycline-based chemotherapy. J. Echocardiogr. 17, 76–83. 10.1007/s12574-018-0394-4 30099714

[B11] EMA. European Medicines Agency (2019). Assessment report. Dexrazoxane-containing medicinal products (EMEA/H/A-31/1275). London, United Kingdom www.ema.europa.eu/docs/en GB/document library/Referrals document/Dexrazoxane 31/WC500120340.pdf Accessed April 13, 2019.

[B12] GBD 2016 Causes of Death Collaborators (2017). Global, regional, and national age-sex specific mortality for 264 causes of death, 1980-2016: a systematic analysis for the Global Burden of Disease Study 2016. Lancet 390, 1151–1210. 10.1016/S0140-6736(17)32152-9 28919116 PMC5605883

[B13] Georgakopoulos P. Roussou P. Matsakas E. Karavidas A. Anagnostopoulos N. Marinakis T. (2010). Cardioprotective effect of metoprolol and enalapril in doxorubicin-treated lymphoma patients: a prospective, parallel-group, randomized, controlled study with 36-month follow-up. Am. J. Hematol. 85, 894–896. 10.1002/ajh.21840 20872550

[B14] Gupta V. Kumar Singh S. Agrawal V. Bali S. T. (2018). Role of ACE inhibitors in anthracycline-induced cardiotoxicity: A randomized, double-blind, placebo-controlled trial. Pediatr. Blood Cancer 65, e27308. 10.1002/pbc.27308 30009543

[B15] Heusch G. (2012). HIF-1α and paradoxical phenomena in cardioprotection. Cardiovasc. Res. 96, 214–215. 10.1093/cvr/cvs145 22822099

[B16] Janbabai G. Nabati M. Faghihinia M. Azizi S. Borhani S. Yazdani J. (2017). Effect of Enalapril on Preventing Anthracycline-Induced Cardiomyopathy. Cardiovasc. Toxicol. 17, 130–139. 10.1007/s12012-016-9365-z 27003392

[B17] Kakia A. Wiysonge C. S. Ochodo E. A. Awotedu A. A. Ristic A. D. Mayosi B. M. (2016). The efficacy and safety of complete pericardial drainage by means of intrapericardial fibrinolysis for the prevention of complications of pericardial effusion: a systematic review protocol. BMJ Open 1, e007842. 10.1136/bmjopen-2015-007842 PMC4716243 26733562

[B18] Kaya M. G. Ozkan M. Gunebakmaz O. (2013). Protective effects of nebivolol against anthracycline-induced cardiomyopathy: a randomized control study. Int. J. Cardiol. 167, 2306–2310. 10.1016/j.ijcard.2012.06.023 22727976

[B19] Luft F. C. (2017). The promise of stromal cell-derived factor-1 in novel heart disease treatments. J. Mol. Med. 95, 821–823. 10.1007/s00109-017-1569-6 28710661

[B20] Potter E. Marwick T. H. (2018). Assessment of left ventricular function by echocardiography: the case for routinely adding global longitudinal strain to ejection fraction. JACC Cardiovasc. Imaging 11 (2), 260–274. 10.1016/j.jcmg.2017.11.017 29413646

[B21] Puddighinu G. D’Amario D. Foglio E. Manchi M. Siracusano A. Pontemezzo E. (2018). Molecular mechanisms of cardioprotective effects mediated by transplanted cardiac ckit+ cells through the activation of an inflammatory hypoxia-dependent reparative response. Oncotarget. 9, 937. 10.18632/oncotarget.22946 29416668 PMC5787525

[B22] Rebecca L. S. Torre L. A. Jemal A. (2018). Global Cancer Statistics 2018: GLOBOCAN Estimates of Incidence and Mortality Worldwide for 36 Cancers in 185 Countries. CA Cancer J. Clin. 68, 394–424. 10.3322/caac.21492 30207593

[B23] Robert S. C.-H. Liu J. E. Yu A. F. (2019). Cardiotoxicity of HER2-targeted therapies. Curr. Opin. Cardiol. 34, 451–458. 10.1097/HCO.0000000000000637 31082851 PMC7313632

[B24] Rohit M. Yeh E. T. H. (2016). Mechanisms of cardiotoxicity of cancer chemotherapeutic agents: Cardiomyopathy and beyond. Can. J. Cardiol. 32, 1–28. 10.1016/j.cjca.2016.01.027 27117975 PMC4921299

[B25] Salzer W. L. Devidas M. Carroll W. L. Winick N. Pullen J. Hunger S. P. (2010). Long-term results of the pediatric oncology group studies for childhood acute lymphoblastic leukemia 1984-2001: a report from the children’s oncology group. Leukemia. 24, 355–370. 10.1038/leu.2009.261 20016527 PMC4300959

[B26] Silber J. H. Cnaan A. Clark B. J. Paridon S. M. Chin A. J. Rychik J. (2004). Enalapril to prevent cardiac function decline in long-term survivors of pediatric cancer exposed to anthracyclines. J. Clin. Oncol. 22, 820–828. 10.1200/JCO.2004.06.022 14990637

[B27] Songbo M. Lang H. Xinyong C. Bin X. Ping Z. Liang S. (2019). Oxidative stress injury in doxorubicin-induced cardiotoxicity. Toxicol. Lett. 307, 41–48. 10.1016/j.toxlet.2019.02.013 30817977

[B28] Tebbi C. K. London W. B. Friedman D. Villaluna D. De Alarcon P. A. Constine L. S. (2007). Dexrazoxane-associated risk for acute myeloid leukemia/myelodysplastic syndrome and other secondary malignancies in pediatric Hodgkin’s disease. J. Clin. Oncol. 25, 493–500. 10.1200/JCO.2005.02.3879 17290056

[B29] Tomlinson L. Lu Z. Q. Bentley R. A. Colley H. E. Murdoch C. Webb S. D. (2019). Attenuation of doxorubicin-induced cardiotoxicity in a human in vitro cardiac model by the induction of the NRF-2 pathway. Biomed. Pharmacother. 112, 108637. 10.1016/j.biopha.2019.108637 30798127

[B30] Turgeon R. D. Kolber M. R. Loewen P. Ellis U. McCormack J. P. (2019). Higher versus lower doses of ACE inhibitors, angiotensin-2 receptor blockers and beta-blockers in heart failure with reduced ejection fraction: Systematic review and meta-analysis. PloS One 14, e0212907. 10.1371/journal.pone.0212907 30817783 PMC6394936

[B31] Wen J. Zhang J. Q. Huang W. Wang Y. (2012). SDF-1α and CXCR4 as therapeutic targets in cardiovascular disease. Am. J. Cardiovasc. Dis. 2, 20–28. 22254210 PMC3257156

[B32] Zamani B. Salehi R. Esfahani A. (2018). Protective effect of carvedilol against anthracycline-induced cardiomyopathy on patients with breast cancer and lymphoma. Int. J. Adv. Med. 5, 16–20. 10.18203/2349-3933.ijam20180061

[B33] Zhang W. Lin M. (2010). e0098 HIF-1α, SDF-1α and VEGF gene expression affected by HIF-1α siRNA in MSCs. Heart. 96, A32–A33. 10.1136/hrt.2010.208967.98

